# Clinical significance of spatiotemporal transcriptional bursting and control

**DOI:** 10.1002/ctm2.518

**Published:** 2021-08-06

**Authors:** Diane Catherine Wang, Xiangdong Wang

**Affiliations:** ^1^ Emergency Medicine Sunshine Coast University Hospital Sunshine Coast Queensland Australia; ^2^ Zhongshan Hospital, Department of Pulmonary and Critical Care Medicine, Shanghai Institute of Clinical Bioinformatics Shanghai Engineering Research for AI Technology for Cardiopulmonary Diseases Shanghai China; ^3^ Jinshan Hospital Centre for Tumor Diagnosis and Therapy Fudan University Shanghai Medical College Shanghai China

**Keywords:** RNA regulation, spatiotemporal, superenhancer, transcriptional bursting, transcriptional control

## Abstract

The rapid development of technologies provides the potential to perform real‐time visualization of transcriptional bursting patterns, superenhancer formation and sensitivity to perturbation, and interactions between enhancers, promoters, and regulators during the burst. The transcriptional bursting‐induced fluctuation can modify cell capacities, cell–cell communications, cell responses to microenvironmental changes, and forms of cell death. A large number of clinical and translational studies describe the existence of heterogeneity among cells, tissues, and organs but mechanism‐based understanding of how and why the heterogeneity exists and how it is formed. The transcriptional bursting, fluctuation, and control determine the development of heterogeneity and optimize cell functions in the cell development and differentiation, contribute to the initiation of cell dysfunction and tumorigenesis in response to environments, and development/evolvement of hyper/hyposensitivity to drugs. Spatiotemporal monitoring of transcriptional bursting and control provides a new insight and deeper understanding of spatiotemporal molecular medicine by integrating the transcriptional positioning and function with cell phenotypes, cell–cell communication, and clinical phenomes.

Transcriptionalcontrol is an important and necessary approach, together with other factors, for regulating gene expression, controlling gene activities, and determining gene functions. Transcriptional control is mainly composed of the pattern of transcriptional bursting, formation of superenhancers, and sensitivity of superenhancers to perturbation. The premature transcription termination serves as one of the molecular mechanisms by which transcriptional control occurs, by forming full‐length transcripts and regulating gene expression, close to the transcription start site characterized by high accumulating, binding, and pausing of RNA polymerase II (RNA Pol II).^1^ A large number of models have been suggested to monitor and predict transcriptional control, including single molecule, single cell, loop, unified theory, stochasticity, enhancer binding, bursting kinetics, and silence‐noise models. The spatiotemporal images at high resolution can visualize nascent transcripts or individual protein molecules, complex natures of transcriptional regulation, real‐time dynamics of transcriptional activities, and distinct kinetics of transcription factors and RNA polymerases. The single‐molecule visualization of transcription in living cells demonstrates image features of transcriptional bursts, upstream regulatory steps of bursting, dynamics and regulation of transcriptional bursting, binding kinetics of transcription factors and enhancer–promoter interactions, and clustering/phase separation of the transcriptional machinery. Of those models, the phase separation model was proposed as a simple model to predict major characters of transcriptional control by number, valency, and affinity of the interacting components and networks between transcriptional regulators and nucleic acids.^2^ The phase separation is a process to form classical membraneless organelles, signaling complexes, cytoskeleton networks, and other supramolecular assemblies. The phase separation model with the aid of computer simulations can present the formation, function, and vulnerability of superenhancers, by comparing the difference between transcriptional bursting patterns and the number of simultaneous bursting genes shared single enhancer. The aim of this Editorial is to call special attentions to the understanding of transcriptional bursting and control, real‐time measuring of bursting kinetics, defining of molecular mechanisms by which the transcriptional bursting is modulated, and prospecting of clinical significance of transcriptional bursting and control.

The transcriptional burst is a process where genes are transcribed during a short period to generate gene‐specific temporal patterns of mRNA synthesis. Transcriptional kinetics were measured by gene‐specific on/off‐switching rates in transcriptional activity and altered by cis‐regulatory DNA elements, as bursting kinetics by mean numbers of mRNAs produced during the bursts are highly gene specific. The transcriptional bursting kinetics of target genes is correlated with the binding of the transcription to chromatin in living cells during sporadic bursts. Donovan et al. integrated the *in vitro* and *in vivo* single‐molecule images and found that the transcriptional factor dwell time is associated with the transcriptional burst size, dependent upon the affinity of the binding site and orders of magnitude by nucleosomes.^3^ Multiple RNA polymerases initiate transcription during one burst as long as the transcription factor is bound to DNA, and bursts terminate the transcription factor dissociation. The occurrence of RNA Pol II recruitment and release from promoter‐proximal pausing needs an independently regulated burst initiation, of which the rate, frequency, and intensity are regulated biological perturbations, rather than the polymerase recruitment rate.^4^ It is possible that the cell may select and optimize the biological behaviors and fates by the magnification of transcriptional fluctuation and can modify cell states for meeting the needs of specific commitments through a post‐transcriptional feedback mechanism. This process is important to maintain and optimize cell functions transmitted through successive generations in cell development and differentiation, initiate cell dysfunction and tumorigenesis in response to environments, and develop/evolve hyper/hyposensitivity to drugs. The early changes of transcriptional burst activity can also initiate the differentiation of terminal effector cells, as one of the critical steps with more complexities.

Transcriptional bursts play an important role in evolution of gene families, cell decision‐making, and environmental adaptation. The transcriptional bursting contributes to multiple copies of the same gene for similar developmental expression profiles, and to different regulatory inputs to individual genes for functional diversification of proteins. Differences in gene expression over shorter times have various transcriptional bursting dynamics and behaviors, which are directly instructed by the upstream sequence.^5^ The intrinsic RNA polymerase on DNA is one of the important transcriptional regulators and influencers, responsible for the formation of transcriptional bursting and the generation of stochastic fluctuations in protein levels through the enlargement or reinforcement of transcriptional bursting. Transcriptional bursts‐induced fluctuations can, furthermore, change cell capacities and fates of cell–cell communications, cell responses to microenvironmental changes, and forms of cell death. The heterogeneity of cell phenomes and degree of stochastic fluctuations are highly dependent upon the transcriptional bursting, associated with kinetics of physical–chemical, chemical–chemical, chemical–biological interactions during the transfer of genetic information and the synthesis of RNA molecules. In addition, the transcriptional bursting is regulated by multiple components like chromatin environments, concentration of transcription factors, and enhancer–promoter interactions.

Of transcriptional burst regulators, RNA Pol II as a 550kda complex with 12 subunits, can modulate the process of transcriptional bursting through binding to the promoter upstream of the target gene with aids of a large number of transcription factors. RNA Pol II shares five subunits with I and III and substantial homology with each other. RNA Pol II C‐terminal domain is composed of 52 tandem heptapeptide repeats in humans, connecting to the polymerase core by binding an 80‐residue linker with RNA Pol II Rpb7 subunit. The length and interaction of RNA Pol II C‐terminal domains can modulate the size and frequency of transcriptional bursting and the efficiency of transcription, enhance the recruitment of initial polymerase to the promoter, and reduce domain release from the binding.^6^

The frequency and strength of transcriptional bursts decide cellular state, while environmental factors‐affected cellular state can alter kinetics of transcriptional initiation and elongation, dependent upon intrinsic and extrinsic noises. For example, Fritzsch et al. found that alternations of transcriptional bursting, extrinsic noise, and multiple alleles of target genes influenced intrinsic noises, probably modulated by histone deacetylases.^7^ The degree of gene transcriptional bursts and intercommunications can affect the expression states and behaviors, leading to the formation of nongenetic transcriptional diversities and heterogeneity and to changes of cell states and sensitivities to external interventions. Cis‐regulatory sequences encode transcriptional burst kinetics through the primary encoding of burst frequency and burst size in enhancers and in core promoters, respectively.^8^ Heterogeneity broadly exists between inter and intracells, tissues, and tumors, and is responsible for the development of cancer reoccurrence, drug resistance, and tumor evolution.^9^, ^10^, ^11^ The formation of cell heterogeneity results from molecule‐to‐molecule variations at RNA levels during bursts of protein production, which is dependent upon states of translational bursting activities and controls the On/Off switch. Notch signaling pathways play a dependent role in the modulation of transcriptional burst during switch‐on, rather than during switch‐off.^12^ It may be important for clinicians to clarify the potential relationship between transcriptional heterogeneity and external interventions, whether the variability of transcriptional bursts and behaviors decides cell responses to or depends upon extracellular environments. This can be a new molecular mechanism to understand the development of drug resistance and a new potential to discover the new strategy of therapy.

Spatiotemporal molecular medicine was recently proposed to integrate spatialization, temporization, severity, and dynamics of clinical phenomes and therapies for diseases, for the improvement of patients’ life quality.^13^ As part of spatiotemporal molecular medicine, the spatiotemporal molecular imaging transintegrates clinical images, pathology, spatial transcriptomes, and visualizing simulations to provide multidimensional information for early diagnosis of the disease.^14^ Spatiotemporal molecular signaling and inherent biochemical stochasticity drive dynamics of gene expression in living cells. The spatiotemporal transcriptional control will provide multifunctional information of gene expression and function, for example, expression, binding, activation of transcriptional activators and repressors, regulation of transcriptional initiation and termination, on/off switch of signaling in pathological conditions, and gene/protein functional development. Spatiotemporal analyses of the core promoter elements and enhancers are important to dynamically monitor transcriptional burst size, frequencies and synergistic effects between TATA boxes and initiators. It will provide new insights to understanding the expression and regulation of spatiotemporal transcriptomics, exploring the *in‐situ* interaction between promoter and burst sizes or enhancers and burst frequencies, and defining cell‐type‐specific gene expression and transcriptional kinetics. The transcriptional burst duration and frequency are dependent upon transcription factor nuclear mobility and bound fraction. Transcriptional cobursting pattern between transcriptional sites is located at proximal and distal positions in the nucleus.^15^ Those data indicate that spatiotemporal patterns of transcriptional bursting, spatiotemporal formation of superenhancers, and spatiotemporal sensitivity of superenhancers to perturbation determine the quality of spatiotemporal transcriptional control. It will provide deeper understanding of spatiotemporal molecular medicine.

In conclusion, the rapid development of technologies provides the potential to perform real‐time visualization of transcriptional bursting patterns, superenhancer formation and sensitivity to perturbation, and interactions between enhancers, promoters, and regulators during the burst. The transcriptional bursting‐induced fluctuation can modify cell capacities, cell–cell communications, cell responses to microenvironmental changes, and forms of cell death. A large number of clinical and translational studies describe the existence of heterogeneity among cells, tissues, and organs with lack of mechanism‐based understanding of how and why the heterogeneity exists and is formed. The transcriptional bursting, fluctuation, and control determine the development of heterogeneity and optimize cell functions in the cell development and differentiation, contribute to the initiation of cell dysfunction and tumorigenesis in response to environments, and development/evolvement of hyper/hyposensitivity to drugs. Spatiotemporal monitoring of transcriptional bursting and control provides a new insight and deeper understanding of spatiotemporal molecular medicine by integrating the transcriptional positioning and function with cell phenotypes, cell–cell communication, and clinical phenomes.
